# Protective Effect of Yinhua Miyanling Tablet on Lipopolysaccharide-Induced Inflammation through Suppression of NLRP3/Caspase-1 Inflammasome in Human Peripheral Blood Mononuclear Cells

**DOI:** 10.1155/2016/2758140

**Published:** 2016-10-03

**Authors:** Jingying Sai, Lingxin Xiong, Jingtong Zheng, Chuangui Liu, Yanjiao Lu, Guoqiang Wang, Yawei Wang, Ting Wang, Xuewa Guan, Fang Chen, Keyong Fang, Chao Zhang, Junying Lu, Xiaotian Zhang, Hailin Zhu, Fang Wang

**Affiliations:** ^1^Department of Pathogeny Biology, College of Basic Medical Sciences, Jilin University, Changchun 130021, China; ^2^Department of Clinical Laboratory, The Second Hospital of Jilin University, Changchun 130021, China; ^3^School of Pharmaceutical Sciences, Jilin University, Changchun 130021, China

## Abstract

Yinhua Miyanling Tablet (YMT), the Chinese formula, has long been administrated in clinical practice for the treatment of acute pyelonephritis and acute urocystitis. In the current study, we aimed to investigate the anti-inflammatory effect of YMT* in vitro* and to evaluate the association between anti-inflammation and innate immune response. Human peripheral blood mononuclear cells (PBMCs) were isolated using Ficoll density gradient centrifugation and then were stimulated by Lipopolysaccharide (LPS). The differential gene expression of inflammation-related genes after drug administration was assessed using PCR array, and the protein levels of differential genes were measured by ELISA and Western blot. The result showed that YMT significantly inhibited the expression of NLRP3, Caspase-1, and the downstream cytokine IL-1*β* and suppressed the production of inflammatory mediators TNF-*α*, IL-6, IL-10, and MCP-1 in a dose-dependent manner compared to the LPS group (*P* < 0.01). The finding indicated that YMT exhibited anti-inflammatory effect* in vitro* by suppressing the NLRP3/Caspase-1 inflammasome, and that may have therapeutic potential for the treatment of inflammatory diseases.

## 1. Introduction

Traditional Chinese medicine (TCM), guided by the theory of traditional Chinese medical science, has been applied for over 5,000 years and is valued for its multitarget and less side-effect properties [1]. Yinhua Miyanling Tablet (YMT) formula is composed of ten traditional Chinese medicines:* Lonicerae Japonicae Flos, Scutellariae Barbatae Herba, Polygoni Avicularis Herba, Dianthi Herba, Pyrrosiae Folium, Clematidis Armandii Caulis, Plantaginis Semen, Lophatheri Herba, Taxilli Herba*, and* Junci Medulla* [2]. YMT has been widely used in acute pyelonephritis, acute urocystitis, and chronic prostatitis for clearing heat and detoxifying and for removing dampness through diuresis for more than a decade [3, 4]. Previous studies reported that phenol extract of* Flos Lonicerae, *the monarch drug in the prescription, exhibited significant antibacterial and anti-inflammatory activities [5–11]. Jin et al. found that phenol extract of* Flos Lonicerae* significantly inhibited inflammatory cell infiltration and protein accumulation and inhibited the production of TNF-*α*, PEG2, and NO in Lipopolysaccharide- (LPS-) induced aqueous humor in rat [12]. Alcohol extract of* Flos Lonicerae* promotes the repair of incision and effectively inhibits the secretion of TNF-*α* and IL-6 and significantly increases the production of IL-10 [13]. However, the anti-inflammatory effect of YMT* in vitro* was not well studied. In addition, despite rich clinical experience, the lack of research in molecular mechanism of action weakens the scientific validity of YMT. Thus, we investigated the anti-inflammatory effect of YMT* in vitro* using modern research methods, including PCR array.

When inflammatory stimulus occurs, innate immunity, as the first defender, recognizes and removes invading microorganisms [14, 15]. Therefore, we hypothesized that innate immunity was involved in the anti-inflammatory effect of YMT. NLRP3 (NOD-LRRs containing pyrin domain 3, also known as cryopyrin or NALP3) inflammasome, including NLRP3, ASC, and Caspase-1, is the most studied complex that is involved in innate immunity. PRR (Pattern Recognition Receptor), expressed in the surface of innate immune cells, recognizes PAMP (Pathogen Associated Molecular Pattern) or DAMP (Damage-Associated Molecular Patterns), then initiates effective innate immune responses, and eventually clears the microorganisms and endogenous molecules released by the host. Inactive pro-Caspase-1 can itself be activated by the NLRP3 inflammasome upon pathogen and damage-associated danger signals; active Caspase-1 degrades pro-IL-1*β* and pro-IL-18, the precursor form of IL-1*β* and IL-18, into the mature cytokines by proteolysis, which initiates the secretion of the cytokines.

In the current study, we aimed to investigate the anti-inflammatory effect of YMT* in vitro* and to evaluate whether YMT had anti-inflammatory effect that was mediated by the regulation of innate immunity.

## 2. Materials and Methods

### 2.1. Test Article and Treatment

Yinhua Miyanling Tablet (YMT) was provided by Jilin Huakang Pharmaceutical Co., Ltd. (Lot number: 140306). Some standard compounds were also used in this study. Chlorogenic acid (Lot number: 110753-201415), Rutin (100080-201408), Hyperoside (111521-201205), Naringin (110722-201312), geniposidic acid (111828-201403), Scutellarin (110842-201508), Luteolin (111520-200504), luteoloside (111720-201408), and Apigenin (111901-201102) were purchased from the National Institutes for Food and Drug Control (Beijing, China). Scutellarein (Lot number: 141128) was purchased from the Sichuan Weikeqi Biological Technology Co., Ltd.

**Figure 1:**
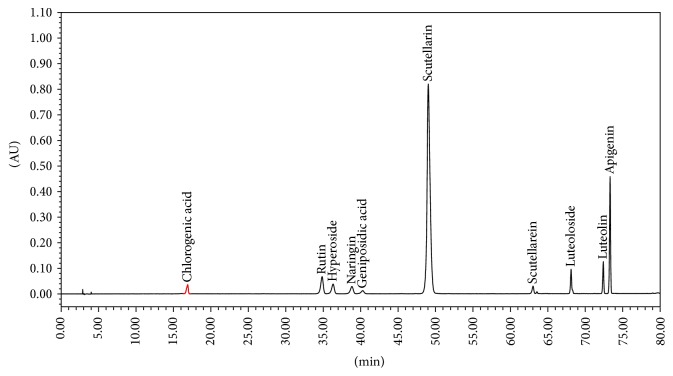
The fingerprints of the mixed standard compounds.

**Figure 2:**
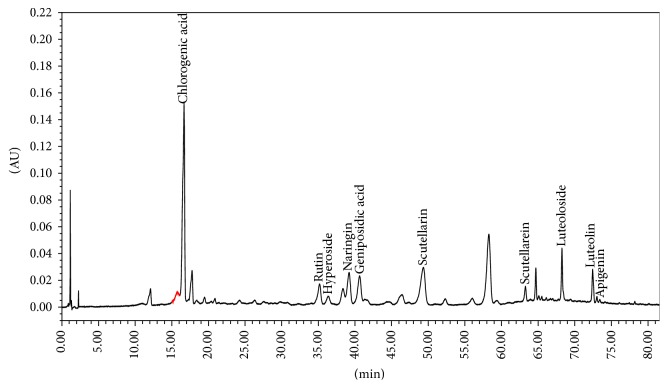
The fingerprints of extract of YMT.

The fingerprint of YMT was further analyzed with a high performance liquid chromatography (HPLC) system (Waters, USA) that consisted of a model 1525 Waters pump, model 2998 Photodiode Array Detector, and Diamonsil C18 Column (5 *μ*m, 250 mm × 4.6 mm). The mobile phase was comprised of acetonitrile (A) and 1% acetic acid in water solvent (B). The gradient mode was as follows: initial 5% A linear gradient to 15% A in 15 min; linear gradient to 17% A in 35 min; linear gradient to 20% A in 55 min; linear gradient to 55% A in 80 min. The flow rate was 1.0 mL/min. The components of YMT were identified by the comparison of the retention time from the chromatograms with those known standards. The fingerprints of the mixed standard compounds and the extract of YMT were shown in Figures 1 and 2.

### 2.2. Materials

LPS and pancreatic enzymes were purchased from Sigma-Aldrich (St. Louis, MO, USA); lymphocyte separation medium was purchased from Jingyang Biological (Tianjin, China); fetal bovine serum (FBS) was obtained from GIBCO (Australia); RPMI-1640 glucose free medium was purchased from Hyclone (Logan, UT, USA); RNeasy Mini Kit, RNase-Free DNase Set, RT^2^ First Strand Kit, RT^2^ SYBR® Green ROX qPCR Mastermix, and Human Inflammasomes PCR Array were obtained from Qiagen (Valencia, CA, USA). ELISA kits of human TNF-*α*, IL-1*β*, IL-6, IL-10, and MCP-1 were purchased from RayBiotech (Atlanta, USA). Anti-NLRP3 and anti-Caspase-1 antibodies were purchased from Abcam (Cambridge, United Kingdom). Secondary antibodies were obtained from Sigma-Aldrich (St. Louis, MO, USA).

### 2.3. Cell Treatment

PBMC was isolated from heparin anticoagulation venous blood via Ficoll density gradient centrifugation method. Then cell viability of isolated PBMC was measured by trypan blue exclusion assay. 50 *μ*L of the cell suspension was mixed with 50 *μ*L of 0.2% trypan blue and incubated for 5 min at room temperature. 10 *μ*L of this mixture was injected beneath the cover slip. The unstained (viable) and stained (dead) cells were counted in a total of 200 cells under microscope (Olympus, Japan). The cell viability was calculated according to the following formula: cell viability = total number of viable cells/(total number of viable cells + total number of dead cells) *∗* 100%. Cells were cultured in RPMI-1640 culture medium containing 10% FBS in a 37°C incubator in the presence of 5% CO_2_. The cells were divided into 5 groups including blank control group (control), LPS-stimulated group (LPS), and three YMT-treated groups including a high concentration group (0.75 mg/mL), a middle concentration group (0.38 mg/mL), and a low concentration group (0.19 mg/mL). The cells in the NC group were cultured in RPMI-1640 culture medium containing 10% FBS. The cells in the LPS group were stimulated with 1000 ng/mL LPS for 19 hours. The cells in the three YMT-administered groups were pretreated with LPS for 1 hour prior to treatment with different concentration of YMT for 18 hours.

### 2.4. Human Inflammasomes PCR Array

Human Inflammasomes PCR Array was performed to evaluate the expression of 84 inflammasomes-related genes. Total RNA was extracted from PBMCs using the RNeasy Mini Kit. An UNIC 2800 UV/VIS Spectrophotometer was used to assess the quantity and quality of the RNA extracts by measuring the absorbance at 260 and 280 nm. Total RNA was purified using an RNase-Free DNase Set. cDNA was generated by reverse transcription of 20 ng of total RNA from each sample using the RT^2^ First Strand Kit and then combined with the RT^2^ SYBR® Green ROX qPCR Mastermix in 96 well arrays. Thermal cycling was performed using an ABI Prism SDS 7300 system (Applied Biosystems). Gene expression was compared using the Ct values and calculated using the ΔΔCt method with normalization to the average expression levels of five common genes (ACTB, B2 M, GAPDH, HPRT, and RPL13A) [16].

### 2.5. Western Blot

After treatment, the cells were collected and lysed in RIPA buffer (Sigma, St. Louis, MO, USA). 25 *μ*g protein per lane was loaded onto SDS-PAGE gels and then were transferred onto nitrocellulose membranes (Millipore Corp., Billerica, MA, USA). The membranes were then blocked and probed using antibodies that were raised against the target proteins, including *β*-actin, NLRP3, Caspase-1, and NLRP1, and were incubated with appropriate secondary antibodies for 2 h. A coloration solution mixture (Beyotime, Jiangsu, China) was added and the immunoreactive bands were determined after exposure to the solution.

### 2.6. Enzyme-Linked Immunosorbent Assay

Protein expression was measured using TNF-*α*, IL-1*β*, IL-6, IL-10, and MCP-1 enzyme-linked immunosorbent assay (ELISA) kits according to manufacturer's instruction.

### 2.7. Statistical Analyses

The data were presented as the means ± SD. Statistical analyses of the data were performed using Student's *t*-test. *P* < 0.05 was considered significant. Statistical comparisons among three groups were performed using one-way ANOVA. These analyses were performed using SPSS 18.0 software (USA) [17, 18].

## 3. Results

### 3.1. LPS-Induced Inflammatory Injury in PBMCs

**Figure 3:**
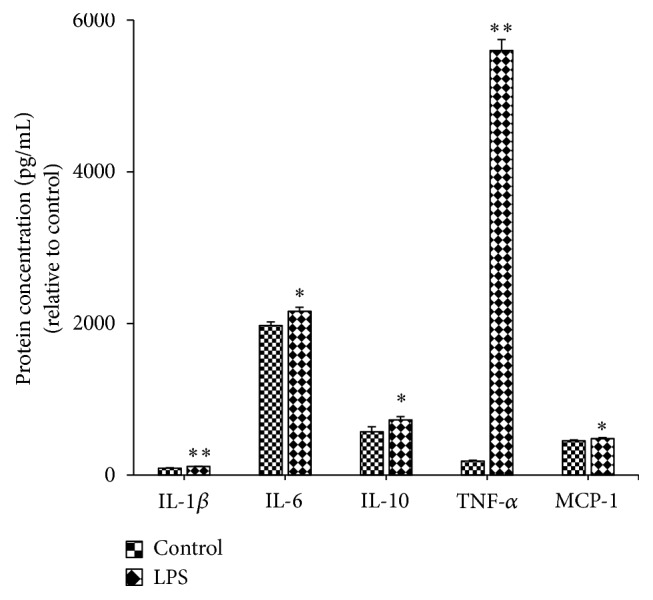
Effect of LPS stimulus on the protein production of IL-1*β*, IL-6, IL-10, MCP-1, and TNF-*α* in PBMC. Control: cells in the NC group were cultured in RPMI-1640 culture medium containing 10% FBS. LPS: cells in the LPS group were stimulated with 1000 ng/mL LPS for 19 hours. The protein levels of IL-1*β*, IL-6, IL-10, MCP-1, and TNF-*α* were determined by ELISA. Results are presented as mean ± standard deviation (SD). ^*∗*^ *P* < 0.05 and ^*∗∗*^ *P* < 0.01 compared to the control group (*n* = 4).

After culture for 18 hours, cell viability of trypan blue stained cell reached 95% after isolation. Our findings showed that after treatment with LPS for 19 hours, the protein expression of TNF-*α*, IL-1*β*, IL-6, IL-10, and MCP-1 was enhanced significantly (Figure 3).

### 3.2. YMT Induces the Differential Regulation of Inflammatory-Related Genes

**Figure 4:**
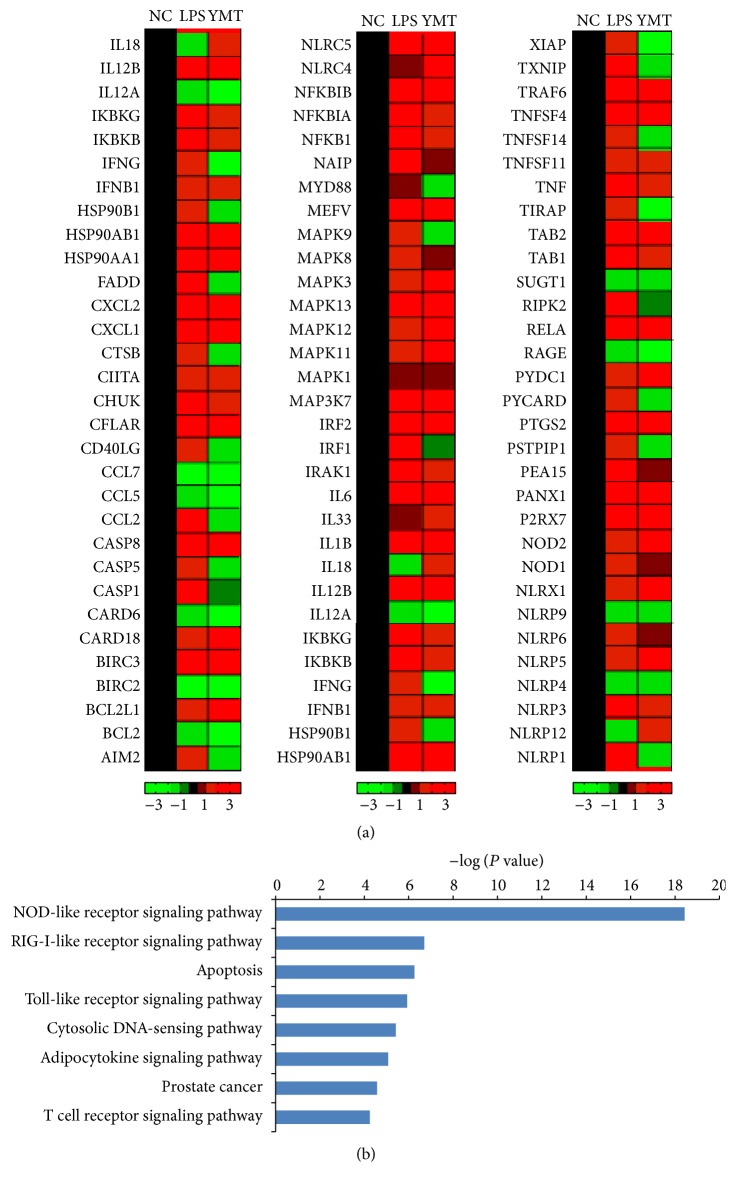
The expression and distribution of differential inflammation-related genes. The results of cluster analysis of 84 inflammation-related genes (a). NC: cells in the NC group were cultured in RPMI-1640 culture medium containing 10% FBS. LPS: cells in the LPS group were stimulated with 1000 ng/mL LPS for 19 hours. YMT: cells in the group were pretreated with LPS for 1 hour prior to the treatment with YMT (0.75 mg/mL) for 18 hours. Black regions represent the gene expression in the NC group. Red regions represent the elevated gene expression compared to the NC group. Green regions represent the decreased gene expression compared to the NC group. The analysis results of differentially expressed genes in KEGG database (b).

We examined the expression of 84 inflammatory-related genes in PBMC from the control group, LPS group, and the YMT-treated groups using the Human Inflammasomes PCR Array. After LPS stimulus, 45 genes were detected with significant change compared to the control group, in which 42 (approximately 93.33%) were upregulated. However, in the YMT groups, we detected 33 differentially expressed genes compared to the LPS group, in which 5 genes were upregulated (Figure 4(a)). 15 genes in the YMT-treated groups (Fold Change > 2) were significantly downregulated by YMT (Table 1). Pathway analysis of differentially expressed genes using DAVID database demonstrated that these genes were involved in NOD-like receptor signaling, RIG-I-like receptor signaling, apoptosis, Toll-like receptor signaling, and cytosolic DNA-sensing (Figure 4(b)).

### 3.3. Effect of YMT on the Protein Expression Levels of NLRP3 and Caspase-1 in PBMC

**Figure 5:**
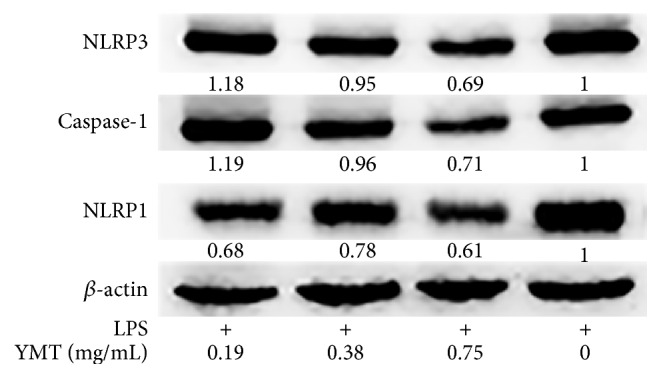
Effect of YMT on the protein expression of NLRP3, Caspase-1, and NLRP1 in PBMC. Cells were pretreated with 1000 ng/mL LPS for 1 hour prior to treatment with different concentration of YMT (0.19, 0.38, and 0.75 mg/mL) for 18 hours. The last group of cells was stimulated with 1000 ng/mL LPS for 1 hour. The proteins were extracted from the cell lysates after treatment. Western blot analyses were performed using NLRP3, Caspase-1, and NLRP1 antibodies. *β*-actin served as the housekeeping control.

To investigate whether YMT-mediated anti-inflammatory effect was regulated by NLRP3/Caspase-1 inflammasome, the levels of the two genes were measured using Western blot. In addition, the level of another differentially expressed gene, NLRP1, was evaluated as well.  Figure 5 showed the levels of NLRP3 and Caspase-1 in YMT-treated groups were downregulated in the study when compared to the LPS group. In addition, the decreased level of NLRP1 in dose-dependent manner was not observed in the investigation (Figure 5).

### 3.4. Effects of YMT on LPS-Induced TNF-*α*, IL-1*β*, IL-6, IL-10, and MCP-1 Production

**Figure 6:**
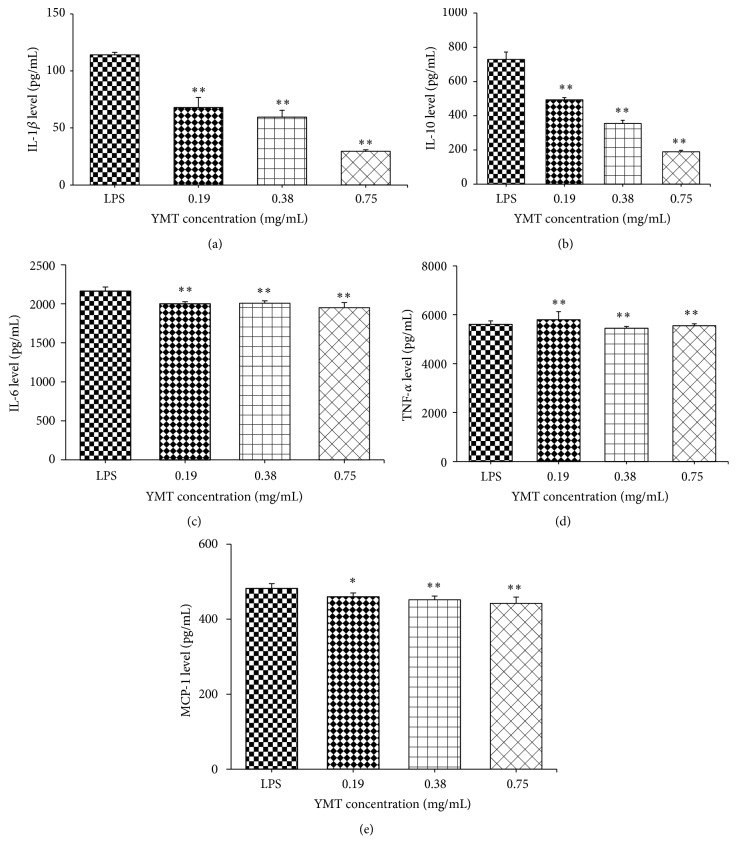
Effect of YMT on the protein levels of IL-1*β*, IL-10, IL-6, TNF-*α*, and MCP-1 induced by LPS in PBMC after 18 hours of treatment. LPS: cells in the LPS group were stimulated with 1000 ng/mL LPS for 19 hours. The remaining groups of cells were pretreated with LPS for 1 hour prior to treatment with different concentration of YMT (0.19, 0.38, and 0.75 mg/mL) for 18 hours. The protein levels of IL-1*β*, IL-10, IL-6, TNF-*α*, and MCP-1 were determined by ELISA. Results are presented as mean ± standard deviation (SD). ^*∗*^ *P* < 0.05 and ^*∗∗*^ *P* < 0.01 compared to the LPS group (*n* = 4).

The suppression of YMT on the production of TNF-*α*, IL-1*β*, IL-6, IL-10, and MCP-1, which may be due to an anti-inflammatory reaction, was determined using ELISA to measure the levels of TNF-*α*, IL-1*β*, IL-6, IL-10, and MCP-1. Treatment with YMT (0.75, 0.38, and 0.19 mg/mL) significantly inhibited LPS-induced TNF-*α*, IL-1*β*, IL-6, IL-10, and MCP-1 production in a dose-dependent manner (*P* < 0.05) (Figure 6).

## 4. Discussion

The important functional role of NLRP3/Caspase-1 inflammasome in the anti-inflammatory effect of YMT on LPS-induced model cells was confirmed in the present study. Our results showed that LPS induced the production of inflammatory mediators IL-1*β*, TNF-*α*, IL-6, IL-10, and MCP-1 as well as the transcription factors NLRP3 and Caspase-1* in vitro* and that YMT significantly inhibited the expression of NLRP3, Caspase-1, and the downstream cytokine IL-1*β* and suppressed the production of inflammatory mediators TNF-*α*, IL-6, IL-10, and MCP-1 in a dose-dependent manner compared to the LPS group (*P* < 0.01).

In previous studies, several herbal drugs comprising* Flos Lonicerae* or the extract of* Flos Lonicerae* were reported to exhibit anti-inflammatory property. Kang et al. found that* in vivo* treatment of ethyl acetate fraction of* Flos Lonicerae* (GC-7101) in experimental gastric ulcer model rats exhibited anti-inflammatory effect by decreased myeloperoxidase activity, NF-*κ*B translocation, and inflammatory cytokines mRNA expression [19]. Cheng et al. treated LPS-activated murine RAW264.7 macrophages with absolute ethanol extract of herbal formula containing* Lonicerae Japonicae Flos* and observed that the inhibitory effect of the extract on the inflammatory mediators was regulated by the NF-*κ*B and MAPK signaling pathways [20]. Similarly, in another work, Cheng et al. investigated the* in vitro* anti-inflammatory activity of herbal formula containing* Lonicerae Japonicae Flos* and concluded that the anti-inflammatory effect was associated with suppression of the IRAK-1/TAK1 and TBK1/IRF3 signaling pathways [21]. Thus, we assumed that YMT may have anti-inflammatory activity as well and then conducted the investigation to confirm it.

LPS stimulation upregulates the expression of the inflammasome related genes, NLRP3, Caspase-1, and IL-1*β* in PBMC from multiple sclerosis compared to healthy controls [22]. In agreement with this finding, in the current study the results demonstrated the upregulation of NLRP3 and Caspase-1 in PBMC from the LPS group compared to the control group (Figure 5) and showed the increased level of IL-1*β*, IL-6, IL-10, MCP-1, and TNF-*α* compared to the control group (Figure 3). Primed bone-marrow-derived macrophages (BMDMs)* in vitro *stimulated with crude extract of LPS produce IL-1*β* and LPS-induced CD8^+^ T cell/adipocyte secrete inflammatory mediators TNF-*α*, IL-6, and MCP-1 as well as the transcription factor NF-*κ*B p 65 [23, 24]. After intraperitoneal injection in C57BL/6 mice with LPS for 6 h, IL-1*β* in peritoneal washes was detected, which confirms the* in vitro* work [23]. LPS, the bacterial endotoxin, binds to the receptor Toll-like receptor 4 (TLR4) and initiates the recruitment of the adaptor myeloid differentiation factor 88 (MyD88) to Toll-interleukin 1 resistance (TIR) domain, resulting in the activation of multiple signaling pathways and the production of proinflammatory mediators [25]. Thus, we chose LPS and PBMC to make inflammatory model to investigate the anti-inflammatory effect of YMT* in vitro*.

We observed the LPS-induced mRNA and protein expression of NLRP3 and Caspase-1, the two components of the NLRP3 inflammasome, was markedly downregulated after YMT treatment in PBMC (Figure 5, Table 1). Oh et al. discovered that the anti-inflammatory activity of methanol extract of* Morus bombycis Koidzumi* was mediated by attenuation of NLRP3 inflammasome activation and regulation of the interferon-*β* receptor signaling pathway in LPS-stimulated RAW264.7 cells and bone-marrow-derived murine macrophages [26]. Sun et al. observed that* Impatiens textori Miq.* extract exhibited the anti-inflammatory effect via inhibition of NLRP3 inflammasome activation in* in vitro* and* in vivo* experimental models and observed decreased amount of Caspase-1 maturation as well [27]. NLRP1 activation leads to the recruitment and activation of the downstream protease Caspase-1 and the secretion of IL-1*β* and IL-18 [28]. Moreover, we did not discover the decreased protein production of NLRP1 despite the suppressed mRNA level after YMT treatment for 18 h (Figure 5). Thus, we concluded that NLRP1 was not involved in anti-inflammatory mechanism of YMT and that the inhibition of NLRP3/Caspase inflammasome was responsible for anti-inflammatory effect of YMT* in vitro*. The NLRP3 inflammasome, including NLRP3, ASC, and Caspase-1, accumulates at the inflammatory sites and activates inactive pro-Caspase-1 when exogenous or endogenous danger signals are detected. Active Caspase-1 maturates the two inflammatory mediators pro-IL-1*β* and pro-IL-18 by proteolysis and promotes their secretion. Via KEGG database analysis, we found that YMT mainly mediated NOD-like receptor signaling pathway and Toll-like receptor signaling pathway, indicating that YMT exerted strong anti-inflammatory activities by enhancing innate immune responses. It is known that binding of LPS to TLR4 activates several intracellular signaling pathways that include the I*κ*B kinase- (IKK-) NF-*κ*B pathway and MAPK pathways, resulting in the expression of IL-1*β*, IL-18, TNF-*α*, and NLRP3 [29]. We noted the downregulation of five NF-*κ*B related genes including RIPK2, RELA, IKBKG, NFKBIA, and CHUK. Among them, RIPK2, RELA, and IKBKG are responsible for encoding and activating the transcription factor, NF-*κ*B, and in contrast NFKBIA and CHUK inhibit the expression of NF-*κ*B. In previous studies, the inhibition of NF-*κ*B signaling pathway was reported to play an important role in the regulation of anti-inflammatory effect [30, 31]. Whether the canonical NF-*κ*B signaling pathway is involved in the anti-inflammatory effect of YMT* in vitro* remains undefined and needs further investigation in the future in order to reinforce the findings of the current study. Inflammation is a multicellular and multifactorial pathophysiological process of many diseases and is involved in a wide range of diseases including asthma, cancer, diabetes mellitus, and pneumonia [32]. Moreover, it is a characteristic of the coexistence of damage and antidamage activities, which causes the inflammatory cellular response and mediator release in response to pathogenic factors [33]. Proinflammatory cytokines induce the production of anti-inflammatory cytokines; on the other hand, anti-inflammatory cytokines suppress the secretion of proinflammatory cytokines rather than function solely, which forms a negative feedback loop and thus enhances innate immunity. IL-1*β*, a downstream protein in the NLRP3/Caspase-1 pathway, cooperates with other cytokines to activate B cells and T cells, induce the production of other inflammatory mediators including TNF-*α* and IL-6, and strengthen the adhesion of leucocytes to endothelial cells. To verify the results obtained in PCR array, we conducted ELISA assay to determine the protein level of inflammation-related genes. The protein production of IL-1*β*, TNF-*α*, IL-6, IL-10, and MCP-1 in supernatant of culture medium decreased significantly after YMT pretreatment in the study (*P* < 0.01) (Figure 6). Under the LPS stimulation, mononuclear macrophage and other inflammatory cells are irritated to produce and release large amount of multiple inflammatory mediators [34], which is consistent with our finding.

## 5. Conclusions

In summary, YMT protects against LPS-induced inflammation in PBMC through the inhibition of the NLRP3/Caspase inflammasome, reducing the production of IL-1*β*, TNF-*α*, IL-6, IL-10, and MCP-1 and suppressing the inflammatory response. The findings demonstrated the strong anti-inflammatory properties* in vitro* and inhibitory mechanism of YMT in NLRP3/Caspase inflammasome. Therefore, the anti-inflammatory ability of YMT may have therapeutic potential for the treatment of other inflammatory diseases in addition to acute pyelonephritis, acute urocystitis, and chronic prostatitis.

## Figures and Tables

**Table 1:** Significantly downregulated inflammation-related genes in PBMC following YMT treatment for 18 h.

Number	Gene name	M/N	Y/M	Description
1	CASP1	2.00	−2.03	Caspase 1, apoptosis-related cysteine peptidase
2	CCL2	2.28	−2.94	Chemokine (C-C motif) ligand 2
3	CHUK	2.54	−2.01	Conserved helix-loop-helix ubiquitous kinase
4	FADD	2.68	−4.40	Fas (TNFRSF6) associated via death domain
5	HSP90AB1	5.74	−2.68	Heat shock protein 90 kDa alpha, class B member 1
6	IKBKG	3.42	−2.03	Inhibitor of kappa light polypeptide gene enhancer in B cells, kinase gamma
7	IRF1	4.96	−5.13	Interferon regulatory factor 1
8	NFKBIA	3.65	−3.33	Nuclear factor of kappa light polypeptide gene enhancer in B cells inhibitor, alpha
9	NLRP1	2.48	−3.41	NLR family, pyrin domain containing 1
10	NLRP3	3.13	−2.17	NLR family, pyrin domain containing 3
11	PEA15	2.55	−2.53	Phosphoprotein enriched in astrocytes 15
12	RELA	4.75	−2.11	V-rel reticuloendotheliosis viral oncogene homolog A (avian)
13	RIPK2	2.37	−2.44	Receptor-interacting serine-threonine kinase 2
14	TNF	6.06	−3.65	Tumor necrosis factor
15	TXNIP	2.68	−3.71	Thioredoxin interacting protein
